# Supplementary material for: Cervical-Vaginal Mucin in Fertility Assessment: CA125 as a Predictor of the Fertile Phase of the Normal Menstrual Cycle
Source: Medicina (Kaunas). 2020 Jun 20;56(6):304. doi: 10.3390/medicina56060304 (PMC7353875; doi:10.3390/medicina56060304)
Supplement: Supplementary file 1 [file medicina-56-00304-s001.pdf]

Table S1. Characteristics of Cycles
Length in days, age, ca125.fsd = CA125 level on FSD (Fertile Start Day), ca125.max = maximum CA125 level.

	subject	cycle	length	age	ca125.fsd	ca125.max	fsd
1	PT1.1	1	24	38	204	869	-5
2	PT1.2	2	26	38	217	513	-4
3	PT2	1	29	39	44	931	-3
4	PT10	1	29	25	84	84	-4
5	PT13	1	31	30	13	87	-7
6	PT14	1	30	31	4	226	-5
7	PT15	1	21	34	12	24	-3
8	PT16.1	1	35	30	28	132	-7
9	PT16.2	2	28	30	38	429	-3
10	PT17.1	1	30	25	10	145	-3
11	PT17.2	2	32	25	5	42	-3
12	PT11.1	2	34	23	9	203	-5
13	PT11.2	3	30	23	8	5740	-5
14	PT18	1	33	30	26	43	-9
15	PT21.1	1	24	30	50	116	-3
16	PT21.2	2	27	30	167	451	-4
17	PT22	1	28	23	4	4	-2
18	PT24	1	30	35	10	99	-11
19	PT26	1	26	31	9	2390	-4
20	PT27	1	27	34	44	67	-6

Table S2.

Estimated Probabilities of Conception (PoC)

	fsd	Daily PoC	PoC
1	-8	0.003	0.000
2	-7	0.014	0.003
3	-6	0.027	0.017
4	-5	0.068	0.042
5	-4	0.176	0.100
6	-3	0.237	0.212
7	-2	0.255	0.268
8	-1	0.212	0.276
9	0	0.103	0.277
10	1	0.008	0.277
11	2	0.035	0.277
